# Maternal Filarial Infection Influences the Development of Regulatory T Cells in Children from Infancy to Early Childhood

**DOI:** 10.1371/journal.pntd.0005144

**Published:** 2016-11-18

**Authors:** Madhusmita Bal, Manoranjan Ranjit, K. Gopinath Achary, Ashok K. Satapathy

**Affiliations:** Division of Immunology, Regional Medical Research Center (Indian Council of Medical Research), Chandrasekharpur, Odisha, India; University Hospital of Bonn, GERMANY

## Abstract

**Background:**

Children born from filarial infected mothers are comparatively more susceptible to filarial infection than the children born to uninfected mothers. But the mechanism of such increased susceptibility to infection in early childhood is not exactly known. Several studies have shown the association of active filarial infection with T cell hypo-responsiveness which is mediated by regulatory T cells (Tregs). Since the Tregs develop in the thymus from CD4+ CD25^hi^ thymocytes at an early stage of the human fetus, it can be hypothesized that the maternal infection during pregnancy affects the development of Tregs in children at birth as well as early childhood. Hence the present study was designed to test the hypothesis by selecting a cohort of pregnant mothers and children born to them subsequently in a filarial endemic area of Odisha, India.

**Methodology and Principal finding:**

A total number of 49 pregnant mothers and children born to them subsequently have been followed up (mean duration 4.4 years) in an area where the microfilariae (Mf) rate has come down to <1% after institution of 10 rounds of annual mass drug administration (MDA). The infection status of mother, cord and children were assessed through detection of microfilariae (Mf) and circulating filarial antigen (CFA). Expression of Tregs cells were measured by flow cytometry. The levels of IL-10 were evaluated by using commercially available ELISA kit. A significantly high level of IL-10 and Tregs have been observed in children born to infected mother compared to children of uninfected mother at the time of birth as well as during early childhood. Moreover a positive correlation between Tregs and IL-10 has been observed among the children born to infected mother.

**Significance:**

From these observations we predict that early priming of the fetal immune system by filarial antigens modulate the development of Tregs, which ultimately scale up the production of IL-10 in neonates and creates a milieu for high rate of acquisition of infection in children born to infected mothers. The mechanism of susceptibility and implication of the results in global elimination programme of filariasis has been discussed.

## Author Summary

Lymphatic filariasis caused by thread like filarial worms involves asymptomatic to acute and/or disfiguring chronic conditions like lymphoedema, elephantiasis and scrotal swelling. Infection occurs when filarial parasites are transmitted to humans through mosquitoes. Adult worms lodge in the lymphatic system and disrupt the immune system that causes the disease. Nonetheless the infection if present during pregnancy, it affects the immune system of the unborn child in such a way that they become more susceptible to infection. But how the immune system of a fetus is affected by the maternal filarial infection is not known. Since regulatory T cells are responsible for development of hyporesponsiveness, a condition that supports the active filarial infection, and develops in thymus at an early stage of the human fetal development, we hypothesized that maternal filarial infection might be affecting the development of Tregs cell. Because Tregs secret IL-10, a regulatory cytokine, we have also measured its level in children born to infected and uninfected mother and correlate it with Tregs. We have observed a significantly high as well as a positive correlation between Tregs and IL-10 levels in children born to infected mother than the children of uninfected mother at the time of birth as well as early childhood indicating that Tregs and IL-10 contribute to immune modulation during pregnancy. Since ongoing MDA excludes pregnant mothers and children below 2 years of age, hence implementation of supervised therapy at the time of adolescent through MDA may help the programme in achieving the target of global elimination of LF by 2020.

## Introduction

Lymphatic filariasis (LF) is a major cause of chronic morbidity in the tropics and sub tropics. According to a recent estimate more than 1.4 billion people across the world are at the risk of infection [1]. To eliminate LF globally by 2020, WHO has introduced annual mass drug administration (MDA) in different endemic countries since one and half decades. But studies have shown that the infection remains highly prevalent among children below five years of age even after several rounds of MDA [2–4]. Here question arises what makes these children more susceptible to infection even though infection levels have come down below threshold in these endemic areas. It is known that besides host genetics and environmental factors, maternal filarial infection plays some role to increase the susceptibility and outcome of the disease. Since pregnancy and early childhood are critical periods during which the inherited immune system of a child is shaped by the environment, the disease outcome in older age is possibly determined both in in-utero and at birth [5]. But it is not exactly known how in-utero exposures to parasite antigens affect immune responses and ultimately the outcome of disease in early childhood. The mechanism of such effects deserves to be explored since our previous findings suggest supervised therapy before pregnancy can reduce the infection rate among children [6].

Induction of regulatory T cells (Tregs) by pathogen is regarded as one of the mechanism of immune evasion in human. It is known that T cell hyporesponsiveness is associated with the active filarial infection, which is partly mediated by regulatory T cells [7]. The immune suppressive capacities of Tregs are due to production of down regulatory cytokines to inhibit inflammatory responses and facilitate the parasite survival [8,9]. Moreover a highly skewed Th2-type cytokine pattern, with a prominent role for the regulatory cytokine interleukin-10 (IL-10) has also been marked in neonates born to helminth-infected mothers [10]. In case of patent filarial infection the state of immune hyporesponsiveness has been observed to be associated with decreased proliferative responses and increased anti-inflammatory cytokines such as IL-10 and TGF-β [8,11]. As the Tregs develop in the thymus at an early stage of the human fetal development from CD4+CD25^hi^thymocytes [12], the question arises that whether maternal infection during pregnancy affects the development of Tregs in children during their early life. Here we have made an attempt to find out the answer by evaluating the infection status, level of Tregs and regulatory cytokine IL-10 in a cohort of children born to filarial infected and non infected mothers.

## Materials and Methods

### Ethics statement

Healthy pregnant women and their offspring born in Khurda District Headquarter Hospital of Odisha, India were enrolled in this mother-child cohort study. The study has received the approval from human ethical committee of the institute with a clause to obtain informed verbal consent from the research participants. The purpose of this research study has been explained in detail to all enrolled mothers in local language in presence of an unbiased witness of the community like Auxiliary Nurse Midwife (ANM) / Accredited Social Health Activist (ASHA) / Anganwadi Workers (AWW). All participants have given face to face oral consent for themselves and their children without a sign consent form to participate over the entire period of study. The name and detailed address of each consent participant has been recorded in data sheet both at the time of enrolment and during follow-up. The oral consent was preferable because (i) the project involves no risk while giving service to the public and benefits to the ongoing LF elimination programme and (ii) linguistic or literacy demands of the written format which requires signature or thumb impression.

### Study design and study participants

This is a cohort study conducted in District Headquarter Hospital of Khurda, Odisha, India, known to be endemic for filarial (*Wuchereria bancrofti*) infections. The district has experienced 10 rounds of MDA with > 85% coverage since 2004 and reported 0.34% Mf in 2013 against 12% in 2004. The pregnant mothers admitted in the hospital for delivery during 2009–2011 without any complications, free from other chronic diseases and belongs to this region have been selected for the study. The pregnant mothers and their subsequently born children enrolled in the study live in 8 adjacent villages. The mother’s age, parity status, levels of formal education, clinical history of filariasis and history of drug consumption in MDA were recorded after enrollment. None of the mothers had signs/symptoms of clinical filariasis at the time of admission. All enrolled mothers have affirmed consumption of anti-filarials distributed during the annual MDA before pregnancy but not during pregnancy since the drugs are not recommended during pregnancy. At the time of delivery blood samples were collected from both mother and cord aseptically and aliquot in different sized tubes to avoid the chance of mislabeling. Serum was separated after centrifugation and stored at—70°C until further use.

Enrolled mothers having healthy full-term children were followed up in a house-to-house visit in the year 2014–15. During follow up along with detailed clinical history 1ml of venous blood sample was collected aseptically from each enrolled mothers and her children. On the basis of the availability of the baseline immunological parameters 49 mother-child pairs were identified for follow-up out of 158 mother-newborn pairs enrolled during 2009–2011. Amongst those 49 children, 28 are within 2–4 years of age and 21 within 4–7 years of age. Infection status of the mother-cord pair at the time of delivery and mother- child during follow-up was determined by diagnosing the presence of microfilaria and/or circulating filarial antigen in the peripheral blood collected at night between 20:30 to 22:30. The Mf (*W*. *bancrofti*) was determined by microscopy by examining the Giemsa stained thick blood smear and CFA was evaluated in serum samples using commercially available Og4C3 antigen detection assay kit (Trop BioMed, Townsville, Australia) following the manufacturer’s instructions.

### Flow cytometry

**Fig 1:**
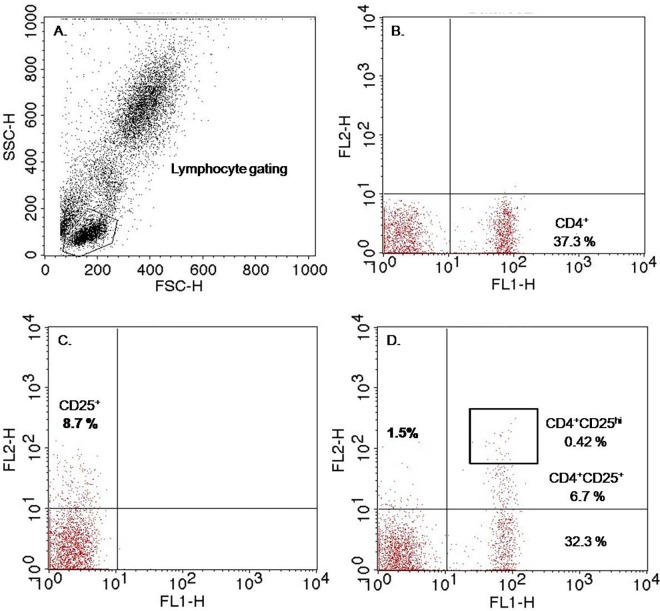
Gating strategy of regulatory T Cells (CD4 cells expressing High CD25 Marker, CD4+CD25^hi^). CD4+ CD25^hi^ cells were characterized by flow cytometry. Fig 1A represents a dot plot showing lymphocyte gating from the PBMC population based on forward and side scatter. Lymphocytes were analyzed by flow cytometry for CD4 and CD25 expression after staining with anti-human CD4-FITC and anti-human CD25-PE. The CD4+ T cells expressing the highest level of CD25+ are considered as T regulatory (Tregs) cells. % of Tregs frequencies (CD4+CD25^hi^) were calculated from total lymphocyte gated. Numbers in each quadrant represent the percentages of that cell type calculated from total lymphocyte gated. Lower right quadrant of Fig 1B represents CD4+cells, Upper left quadrant of Fig 1C- represents CD25+cells, Upper right quadrant of Fig 1D represents CD4 + CD25+ cells and Box represent CD4 cells expressing high levels of CD25 marker.

The identification of Tregs (CD4+ and CD25^hi^ T-cells) was determined by using fluorescently labeled antibodies specific to surface markers (CD4 and CD25). Briefly, 50 μl of heparinized blood collected from mother, cord and children were incubated in dark with 10 μl of anti-human CD4-FITC (BD-Bioscience), anti-human CD25-PE (BD-Bioscience) for 30 minutes at 4°C followed by addition of 2ml of lysing solution and incubation for 10 minutes at room temperature. The samples were then centrifuged at 250 X g for 10 mins and cell pellets were washed twice with 2 ml of sheath fluid (BD Bioscience). Finally the cell pellets were re-suspended in 0.5 ml of sheath fluid and subjected to flow cytometric analysis. Data were acquired by using BD FACS calibur flow cytometer and analyzed using cellquest pro software. The gating strategy for Tregs (CD4+CD25+ ^hi)^ cells is displayed in Fig 1.

### Cytokine analysis

The level of IL-10 was determined using IL-10 assay kit (Sigma Aldrich, USA) according to the instructions supplied by the manufacturer. Briefly, 100 μl of plasma and standards were added to each well of the antibody coated ELISA plate. The plate was sealed and incubated overnight at 4°C with gentle shaking followed by (i) 4 x wash with wash buffer and incubation with 100 μl of biotinylated detection antibody for 1 hour at room temperature, (ii) 4 x wash and incubation with 100 μl of HRP—streptavidin conjugate for 45 minutes at room temperature and (iv) 4x wash and incubation with 100 μl of colorimetric TMB reagent for 30 minutes. Finally50 μl of stop solution of 0.2M H_2_SO_4_was added and read in ELISA reader at 450 nm.

### Statistical analysis

The statistical analysis was performed using GraphPad Prism software (version 4). Mann-Whitney test was used to analyze the difference between two groups of unpaired data and Wilcoxon signed rank test for paired data. Fisher's exact test was used to compare the difference of proportions between two groups. Kruskal-Wallis test with the addition of Dunn test was used to analyze the difference between more than two independent groups. The associations between Tregs and IL-10 levels were analyzed using Pearson’s correlation analysis. The level of significance was set at 0.05.

## Results

**Fig 2:**
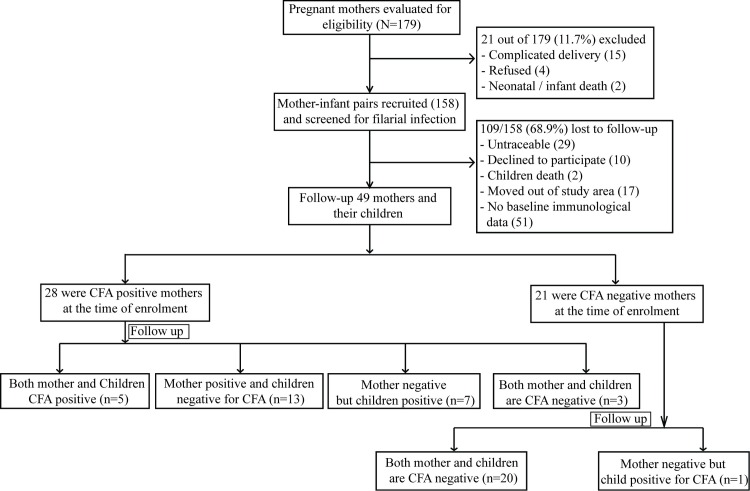
The flow diagram of the cohort study.

The summary of the enrolment and follow up of participants is depicted in Fig 2. A total number of 179 pregnant women admitted to hospital for delivery from July 2009 to July 2011 were evaluated for inclusion in this study. Twenty one (11.7%) of them was excluded because of complication during delivery or infant death or unwillingness. Finally 158 mother-new born pairs were enrolled for the study. At the time of enrolment 11.8% of the mother were microfilariae positive (3–210 per 60μl blood), whereas 44.5% of pregnant mothers were CFA positive (GM: 1925, range: 630–16596). Interestingly, 24.5% of infected mothers have shown transplacental transfer of filarial antigen to their cord, while none of the cord blood from CFA negative mother was CFA positive. Similarly the cord blood of neither CFA +ve nor CFA–ve mother was positive for Mf. During the study period total 109 mother-child pairs have been dropped because they are either non traceable, decline to participate, death of the children, moved out of study area or non availability of immunological parameter. Finally 49 pregnant mothers and their subsequently born children have been followed up during 2014-15.The mean duration of follow-up was 4.4 years (range, 2–7 years).

The characteristics of follow-up mothers and children have been described in Table 1. Amongst 49 follow up mothers 28 were CFA positive and 21 were CFA negative at the time of recruitment. Of the total 28 CFA positive mothers, only 3 were Mf positive at the time of enrollment. All of the study participants were living in rural areas and majority of them (83.3%) were house wives by occupation with primary level of school education (77.5%). Except filarial infection status, no difference was noticed in terms of age in years, multiparity status and educational level among the CFA +ve and CFA-ve mothers during follow-up. Amongst the CFA positive (n = 28) follow-up mothers, 18 mothers are still harbouring filarial infection (CFA +ve but Mf–ve) without any clinical symptoms of filariasis, 4 mothers have cleared CFA but have developed acute symptoms of filariasis (episodic attack of fever associated with inflammation of lymph nodes and lymphatics of legs/arms) and 6 mothers have cleared CFA without development of any clinical symptoms of filariasis. Whereas none of the CFA negative mothers had acquired filarial infection or developed any clinical sign/symptoms of filariasis.

**Table 1:** Characteristic of CFA positive and CFA negative mothers and their children in the study during follow-up

Participant Mothers	*CFA positive	*CFA negative	P value
Number of subjects	28	21	
**Characteristics of mother**			
Age in years, median (range)	27 (22–35)	25 (21–36)	P = 0.33
Multiparity status, n (%)	13 (46.4)	11 (52.38)	P = 0.776
Occupation (House wives) n (%)	24 (85.7)	17 (80.9)	P = 1.0
Education (Primary School) n (%)	22 (78.5)	16 (76.1)	P = 1.0
Microfilariae status n (%)	0.0	0.0	
Clinical sign and symptom of Filariasis n (%)	4 (14.2)	0.0	NA
Circulating filarial antigen +ve n, (GM, Range)	18 (245.3, 127–7762)	0.0	NA
**Characteristics of Children**			
Age in years, median (range)	4 (2–7)	3(2–7)	P = 0.40
Female n (%)	12 (42.85)	10(47.61)	P = 0.778
Microfilaraemia	0.0	0.0	
Circulating filarial antigen +ve, n(%),(GM, Range)	12 (42.8)(144, 120–223)	1 (4.7)(124)	P = 0.003
Clinical sign and symptom of filariasis n (%)	0.0	0.0	

*Status of mother at the time of enrolment, NA: Not applicable

Out of 28 children born to the infected mothers, 12 (42.8%) children have acquired filarial infection and become CFA positive. In contrast one of the children (1/21, 4.7%) born to the uninfected mothers has acquired filarial infection and become CFA positive. (OR = 15, 95% CI: 1.75–127.9, Z = 2.47, p = 0.013). Amongst the infected children 7 children were in the 2–4 years of age and 6 children were in 5–7 years of age. Out of the 12 CFA positive children 5 were from mothers who continued to be CFA positive where as 7 were from mothers those cleared CFA. While analyzing the infection status of cord of these 28 children it was observed that 21.4% (6/28) of them were CFA positive. Amongst those 6 cords positive children only 2 have become CFA positive during follow up. Statistically no significant difference (p = 0.67) was observed in acquiring infection among children born from CFA +ve mothers having CFA +ve (2/6, 33.3%) and CFA-ve (10/22, 45.4%) cord at the time of delivery. Interestingly none of the cord from uninfected mother was CFA positive at the time of enrollment. Also none of the children born to either infected or uninfected mother have detectable microfilariae and/or with any clinical signs/symptoms of filariasis. Besides presence of CFA no difference was observed in age, gender in children born to infected and uninfected mother. Based on the presence/absence of CFA in mothers and children during follow-up, the children of CFA positive mothers have been divided into 4 sub-groups i.e. group I: both mother and child are CFA positive (M+Ch+, n = 5), group II: mother positive but child negative for CFA (M+Ch-, n = 13), group III: mother negative but child positive (M- Ch+, n = 7) and group IV: both mother and child negative for CFA (M- Ch-, n = 3).

**Fig 3:**
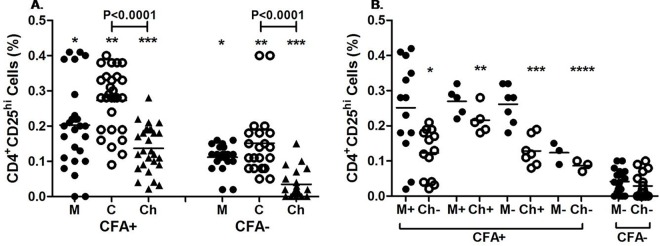
Expression profile of Tregs (CD4+CD25^hi^) cells in mother and cord at the time of enrollment and in children during follow-up. Each dot represents an individual’s frequency of Tregs and lines represent the mean values. **A**. Treg frequency of CFA+ve (n = 28) and CFA-ve (n = 21) mother (M) and their cord (C) at the time of delivery and in children (Ch) during follow up. CFA +: mothers CFA +ve at the time of enrolment. CFA-: Mothers who were CFA-ve at the time of enrolment.* p = 0.016, **p<0.001, ***p<0.0001 **B**. Treg frequency of CFA+ve and CFA-ve mother and their children (Ch) at the time of follow up. M+Ch+: mother and children CFA+ve (n = 5), M+Ch-: mother CFA+ and children CFA-ve (n = 13), M-Ch+: mother CFA-ve and children CFA+ve (n = 7), M-Ch-: mother and children are CFA-ve (n = 3). M-Ch-: born from uninfected (CFA–ve) mother (n = 20).*p<0.0001, **p = 0.0008, ***p = 0.0003, ****P = 0.02 as compared to children (M-Ch-) of CFA–ve group.

The expression of Tregs in infected mother–cord pairs was significantly high as compared to mother-cord pairs of uninfected mother (mother: p = 0.016, cord: p<0.001). Similarly Tregs cell expression was significantly high (p < 0.0001) in children born to enrolled CFA positive group of mothers in comparison to children born to enrolled CFA negative group of mothers (Fig 3A). Further we have observed a decreasing trend in the level of Tregs in children born to both infected and uninfected mother as compared to the cord blood (p <0.0001 for CFA+ve and p < 0.0001 for CFA-ve).

To evaluate the impact of maternal infection on development of Tregs in children during their early childhood, we have analyzed the Tregs in mothers as well as children born to two groups i.e. CFA positive and CFA negative group during follow up. Irrespective of the CFA status of mother at the time of follow-up, Tregs cells were significantly high (p = 0.01) in mothers who were CFA positive at the time of enrollment compared to enrolled CFA negative mothers (Fig 3B). But no significant difference (p = 0.14) in Tregs cell expression was observed among mothers of four different subgroups belonging to CFA positive group. Whereas significantly high Tregs cell expression was observed between these four subgroups of mothers compared to CFA–ve group mothers (M+Ch-vs M-Ch-: p<0.001,M+Ch+ vs M-Ch-: p = 0.0008,M-Ch+ vs M-Ch-:p = 0.0001, M-Ch-vs M-Ch-:P = 0.01).Children born to four sub-groups of CFA positive mothers showed significant (p = 0.01) difference among themselves. Further, children born to these four subgroups of mothers had higher levels of Tregs expression than children born toM-Ch-of CFA -ve mother ((M+Ch-vsM-Ch–:p<0.0001,M+Ch+ vs M-Ch-:p = 0.0008,M-Ch+ vs M-Ch-: p = 0.0003, M-Ch-Vs M-Ch-:p = 0.02). On the other hand one of the children born to CFA–ve mother has acquired filarial infection during follow-up.with 0.15% of Tregs expression.

**Fig 4:**
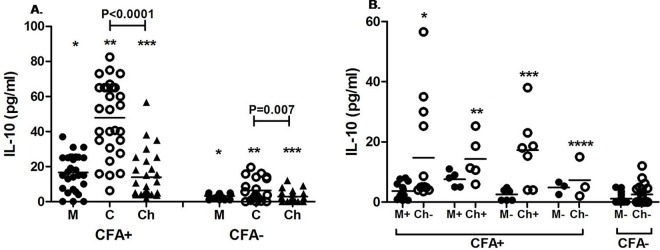
Plasma level of IL-10 in CFA +ve and CFA-ve mothers and their children at the time of delivery and during follow-up. Each dot represents an individual’s IL-10 level and lines represents the mean values. **A**. IL-10 level of CFA+ve (n = 28) and CFA-ve (n = 21) mother (M) and their cord (C) at the time of delivery and in children (Ch) during follow up. CFA +: mothers CFA +ve at the time of enrolment. CFA-: Mothers who were CFA-ve at the time of enrolment.* p<0.0001, **p<0.0001, ***p<0.0001. **B.** IL-10 levels in mother and their children at the time of follow up. M+Ch+: mother and children CFA+ve (n = 5), M+Ch-: mother CFA+ and children CFA-ve (n = 13), M-Ch+: mother CFA-ve and children CFA+ve (n = 7), M-Ch-: mother and children are CFA-ve (n = 3). M-Ch-: children born from uninfected (CFA–ve) mother (n = 20).*p = 0.001, **p = 0.002, ***p = 0.002, ****p = 0.03 as compared to children (M-Ch-) of CFA–ve group.

We have quantitatively assessed the level of IL-10, the hallmark cytokine for regulatory response, in plasma of cord blood as well as children born to infected and uninfected mothers to evaluate role differentiated T helper cell subsets in filarial infection. At the time of enrollment level of IL-10 was significantly higher in mother as well as cord blood of CFA positive mothers as compared to cord and mother of CFA -ve group (mother: p<0.0001, cord: p<0.0001) as shown in Fig 4A.Further a decreasing trend in level of IL-10 has been marked in children compared to cord (p < 0.001 for CFA+ve and p = 0.007 for CFA-ve group). Similarly during follow up significantly higher level of IL-10 was observed in CFA +ve mother as well as their children in comparison to CFA–ve mothers and their children (p<0.0001for mothers, p<0.0001 for children). However when the comparisons were made between the four subgroups of mothers as well as children belonging to the CFA +ve mothers, no significant difference was observed in IL-10 level (p = 0.07 for mothers, p = 0.5 for children) among them (Fig 4B).But IL-10 level in the subgroup of enrolled CFA positive mothers was significantly higher compared to enrolled CFA negative mothers during follow up (M+Ch- vs M-Ch–: p = 0.002,M+Ch+ vs. M-Ch- p:0.002,M-Ch+ vs. M-Ch-: P = 0.013, M-Ch-vs. M-Ch-: P = 0.017). More than that IL-10 level was significantly higher in children born to all four sub-groups of CFA positive mothers than born to CFA negative mother as evident in Fig 4B (M+Ch- vs M-Ch–: p = 0.001, M+Ch+ vs M-Ch-: p = 0.002, M-Ch+ vs M-Ch-: p = 0.002, M-Ch- vs M-Ch- p = 0.03). One child born to CFA–ve mothers acquired infection and having IL-10 level of 9 pg/ml.

**Fig 5:**
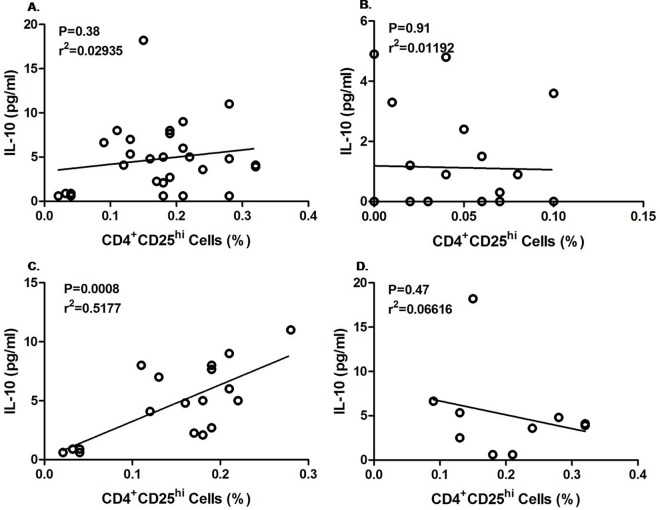
Correlation between Tregs (CD4+CD25^hi^) with IL-10 level in enrolled CFA+ve and CFA–ve mothers during follow up. Each dot represents value from a single subject while the solid lines represent the regression lines. Values of p and r derive from Pearson’s correlation analysis. A. enrolled CFA+ve mothers (n = 28, p = 0.38, r^2^ = 0.029) and B. Enrolled CFA–ve mother (n = 21, p = 0.91, r^2^ = 0.012). C. Enrolled CFA +ve mothers who remained CFA+ve during follow up (n = 18, p = 0.008, r^2^ = 0.518). D. Enrolled CFA+ve mothers who became CFA-ve during follow up (n = 10, p = 0.47, r^2^ = 0.066)

**Fig 6:**
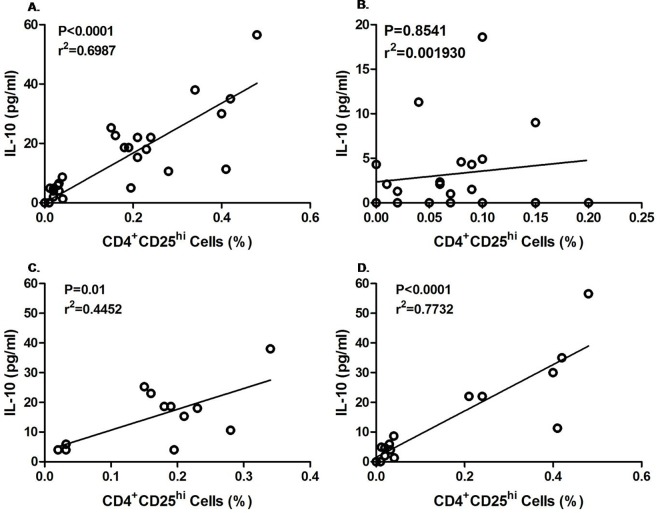
The relationship between regulatory Tregs (CD4+CD25^hi^) with IL-10 level of children born to mother who were CFA +ve and CFA–ve at the time of enrolment. Each dot represents value from a single subject while the solid lines represent the regression lines. Values of p and r derive from Pearson’s correlation analysis. (A) Children of enrolled CFA +ve mother (n = 28, p< 0.0001, r^2^ = 0.6987) (B) children of enrolled CFA-ve mother (n = 21, p = 0.8541, r^2^ = 0.001930).(C) CFA +ve children born to enrolled CFA+ve mother (n = 12,p = 0.01, r^2^ = 0.445) (D) CFA-ve children born to enrolled CFA+ve mother (n = 16, p<0.001, r^2^ = 0.7732).

To find out the effect of Tregs cells on IL-10 secretion in infected and uninfected mother as well as their children, a correlation was made between percentage of CD4+CD25^hi^ cell expressions and IL-10 level during the follow up. As shown in Fig 5A and 5B, no significant correlation was observed between Treg and IL-10 of enrolled CFA+ve (p = 0.38, r2 = 0.029) and CFA–ve mother (p = 0.91, r2 = 0.012) during follow up. However when we differentiate the CFA+ve and CFA-ve group of enrolled CFA+ve mother during follow-up, a highly significant positive correlation was observed among the CFA +ve mothers (p = 0.0008, r2 = 0.518; Fig 5C) in contrast to CFA-ve mothers (p = 0.47, r2 = 0.066, Fig 5D). From Fig 6A, it is evident that a significant positive correlation (p< 0.0001, r^2^ = 0.6987) exists between IL-10 level and Tregs in children of infected mothers. In contrast no correlation was marked between IL-10 level and Tregs in children born to CFA negative mothers (Fig 6B, p = 0.8541, r^2^ = 0.001).Moreover there was no difference in Treg and IL-10 correlation between CFA+ve and CFA-ve children born to enrolled CFA+ve mothers (CFA+ve: p = 0.01, r2 = 0.445; CFA-ve: p<0.001, r2 = 0.7732) as shown in Fig 6C and 6D).

## Discussion

The current study reveals that maternal *W bancrofti* infection during pregnancy up- regulates the production of Tregs and IL10 in offspring from infancy to early childhood and children born to infected mothers are at greater risk of acquiring filarial infection than children born to uninfected mothers. Further, evaluation of cord blood response and their correlation with infection status of children born to infected mothers suggests that in-utero sensitization rather than transplacental transfer of filarial antigen leads to increased susceptibility to filarial infection after birth.

Presence of parasitic infections during pregnancy is known to influence the immune system of an unborn child directly, through transfer of parasites or antigens across the placenta. As a consequence the neonates born to the infected mother become more susceptible to infection [2, 13, 14]. We have observed filarial antigen in 21.4% (6/28) of the cord blood of infected mothers but 42.8% of children born to them have acquired filarial infection which is double the figure of antigenemia of cord blood. This finding is supported by the previous work which showed that prenatal filarial specific immune tolerance as a consequence of active maternal filariasis increased the risk of infection during the first 7 years after birth [15]. Further, out of the 6 CFA positive cord only two children have acquired infection during follow up indicating no significant difference in acquiring infection between children born with CFA positive and CFA negative cord (from CFA positive mother).Therefore, it is speculated that early priming due to in-utero exposure rather than the transplacental transfer of filarial antigen is playing the key role towards disease susceptibility. Conversely, children born to infected mother but have not acquired infection during the childhood even though living in same endemic area may be due to heterogeneity in exposure to infective larvae, co-infection with malaria and geo-helminths that could bias T cell cytokine response and differences in genetic makeup [15].

Initially it was thought that filarial infections profoundly suppress the T-cell-proliferative and IFN-γ responses because of Th2 bias. Though these infections undoubtedly elicit Th2 cells, but recent studies show expansion of regulatory T-cell population while maintaining the hyporesponsive state in filariasis [16]. Epidemiological evidence suggests that in-utero sensitization results down-regulated responses among the offspring, on encountering the homologous antigen which may be due to bias in the fetal and neonatal immune response towards the development T regulatory cell. Moreover filaria-associated Tregs has been demonstrated to modulate the T and B cell proliferation and polarized cytokine production by effector T cells in microfilaraemics [7, 14].In the present study, significantly high level of IL-10 and Tregs cell from infancy to early childhood signifies their role towards the disease susceptibility. Recently some other studies have revealed that modulated/ regulated T cell responses associated with patent filarial infection reflects expansion of Tregs that include both Tregs induced in peripheral circulation and the thymus-derived Tregs [11, 17]. Further filarial infection during pregnancy leads to an expansion of functionally active regulatory T cells that keep Th1 and Th17 in check [18]. In the present study significantly high expression of Tregs in children born to infected mothers is at par with the findings of others and the high level of CD4+CD25^hi^ cells in cord blood of infected mothers supports our hypothesis that regulation of Tregs cells start from the time of early priming during pregnancy. Contribution of such regulatory network towards hyporesponsiveness has been well documented during an in vitro study in newborns of filaria infected mothers [19]. Further, studies have shown that in utero stimulation with helminth-derived antigens divert fetal immunity towards Th2 responses and/or lead to anergy or tolerance [20, 21]. Since Treg cells produce regulatory cytokine IL-10 that modulates the entire repertoire (Th1/Th2/Th17) of CD4+ effector cell responses indiscriminately in filariasis that limits the Th1 response [22,23],we have evaluated the level of IL-10 in two groups of children to draw the functional relationship with Tregs. In our earlier study, cord blood from filarial infected mother exhibited decreased production of IFN-γ (Th1) response and increased production of IL-10 (Th2) indicating that immune responses have already been skewed towards Th2 type of response at the time of birth [24]. In addition the high level of T- regulatory cells and increased production of IL-10 in cord blood of infected mothers could down regulate inflammatory responses and create a susceptible environment for the parasite to grow. Similar to our observation, in a cross sectional study conducted in Kenya by Malhotra and others have found that maternal filarial infection increases childhood susceptibility to *W*. *bancrofti* and skews filaria-specific immunity toward a Th2-type cytokine response [25]. In the present study significantly high level of IL-10 in children born to infected mothers in comparison to children born to uninfected mother emphasizes that in utero sensitization down regulate the immune response in children since the time of birth. Though some of the children born to infected mother are free from infection during follow up yet they maintain high level of Treg and IL-10 which is in agreement with the previous work that shows that helminth-specific T cell immunity acquired in utero is maintained until at least 10 to14 months of age in the absence of infection [26]. In case of CFA-ve group of mothers who are still CFA-ve within the follow-up groups could be then classified as endemic normal as there is no record of infection prior to the survey. The low level of IL-10 in this group suggests that endemic normals have specific immune profile preventing filarial infection. Though the focus on this immunomodulation during helminth infections has been on IL-10, yet contributions of natural T regulatory cells (nTregs) appear to be significant [27, 28] in this context of our study.

A recent study in India has shown that frequencies of regulatory T cell markers were higher in asymptomatic microfilaremics and/or circulating filarial antigen positive subjects than in patients with chronic pathology. It also suggests a more prominent regulatory role of IL-10 producing Tregs [7, 17]. Though in the present study it was not possible to measure the IL-10 producing Tregs still a strong association of Tregs and IL-10 was observed in children born to filarial infected mother during their early childhood. It was also observed that expression of Treg and production of IL-10 in CFA+ve and CFA-ve children born to CFA+ve mothers do not differ from each other. This indicates that in utero priming determines the Treg and IL-10 production independent of acquisition of infection in later life. These findings supports the notion that immunologic memory established by priming of prenatal T cells with antigens that pregnant women encounters the infection that persists from gestation to childhood. This might be the cause of high incidence of infection among the younger age children (2–4 years old) in this cohort as observed by others [3, 15, 25, 26]. From this we can speculate that increased level of Tregs and high production of IL-10 initiates a cascade of hyporesponsive mechanism in children from the time of birth that down regulates the inflammatory responses and lead to a Th2 type of response so as to make them susceptible for parasite survival and ultimately determines the disease outcome in children.

The obvious limitation of our study is small sample size corresponding to both children born to infected and uninfected mothers. Albeit by drawing correlation we can interpret that Tregs in offspring from filarial infected mothers influence the IL-10 production as described in adults. When analyzing regulatory T cells, the measurement of transcription factor FoxP3, CD49b and LAG-3 markers for Treg and Tr1 cell population and intracellular FACS antibodies such as IL-10 in IL-10-producing Tregs were not possible due to poor resource which might have been useful to analyze the functional relationships between their number and mechanism of action.

## Conclusion

In conclusion we can state that maternal filarial infection during pregnancy increases the susceptibility of children to infection by immune priming through expression of Tregs as well as regulatory cytokine IL-10. The high incidence of infection among the younger children even after 10 rounds (2014) of MDA in this area is due to high rate of Mf among pregnant women during 2009. While the cause of high Mf rate among the pregnant women might be due to low compliance because of social customs or back to back pregnancy. Hence the present findings relates to a greater impact on mass treatment programs aimed at elimination of transmission of *W bancofti* infection. To prevent the prenatal immune priming and tolerance supervised therapy can be introduced at the child bearing age of the women, so that they can be free from infection by the time of pregnancy and, thus, decrease the risk of infection during childhood. Implementation of such strategy will help the programme in achieving the target of global elimination of LF by 2020.

## Supporting Information

S1 Checklist STROBE Checklist (DOC)

## References

[1] Rebollo MP, Bockarie MJ. Toward the elimination of lymphatic filariasis by 2020: treatment updates and impact assessment for the endgame. Expert Rev Anti Infect Ther.2013; 11: 723–31. 10.1586/14787210.2013.811841 23879610

[2] Bal MS, Mandal NN, Das MK, Kar SK, Sarangi SS, Beuria MK. Transplacental transfer of filarial antigens from *Wuchereria bancrofti*-infected mothers to their offspring. Parasitol. 2010; 137: 669–673. 10.1017/S0031182009991478 19849889

[3] Bal MS, Beuria MK, Mandal NN, Das MK. Antigenemia in young children living in *Wuchereria bancrofti* endemic areas of Orissa, India. Trans R Soc Trop Med Hyg. 2009; 103: 262–65. 10.1016/j.trstmh.2008.08.006 18809193

[4] Mandal NN, Bal MS, Das MK, Achary KG., Kar SK. Lymphatic filariasis in children: age dependent prevalence in an area of India endemic for *Wuchereria bancrofti* infection. Trop Biomed. 2010; 27(1): 41–46. 20562812

[5] Yenny Djuardi Y, Supali T, Wibowo H, Kruize YCM, Versteeg SA, Ree RV, et al The development of TH2 responses from infancy to 4 years of age and atopic sensitization in areas endemic for helminth infections. Allergy Asthma Clin Immunol. 2013, 9:13 Available 10.1186/1710-1492-9-13 23566643 PMC3635885

[6] Bal M, Sahu PK, Mandal N, Satapathy AK, Ranjit M, Kar SK. Maternal Infection is a Risk Factor for Early Childhood Infection in Filariasis. PLoS Negl Trop Dis. 2015; 9(7): e0003955 10.1371/journal.pntd.0003955 26225417 PMC4520468

[7] Wammes LJ, Hamid F, Wiria AE, Wibowo H, Sartono E, et al Regulatory T cells in human lymphatic filariasis: stronger functional activity in microfilaremics. PLoS Negl Trop Dis. 2012; 6: e1655 10.1371/journal.pntd.0001655 22666510 PMC3362610

[8] Malhotra I, Dent A, Mungai P, Wamachi A, Ouma JH, et al Can Prenatal Malaria Exposure Produce an Immune Tolerant Phenotype?: A Prospective Birth Cohort Study in Kenya. PLoS Med. 2009; 6. 10.1371/journal.pmed.1000116 PMC2707618 19636353

[9] Kurtis JD, Higashi A, Wu H-W, Gundogan F, McDonald EA, et al Maternal Schistosomiasis japonica is associated with maternal, placental, and fetal inflammation. Infect Immun. 2011; 79: 1254–1261. 10.1128/IAI.01072-10 21149589 PMC3067505

[10] Maizels RM, Yazdanbakhsh M. Immune regulation by helminth parasites: cellular and molecular mechanisms. Nat Rev Immunol. 2003; 3: 733–744. 10.1038/nri1183 12949497

[11] Metenou S, Nutman TB. Regulatory T cell subsets in filarial infection and their function.Front Immunol. 2013; 4: 1–8. 24137161 10.3389/fimmu.2013.00305 PMC3786323

[12] Peterson RA. Regulatory T-cells: diverse phenotypes integral to immune homeostasis and suppression. Toxicol Pathol. 2012; 40: 186–204. 10.1177/0192623311430693 22222887

[13] Achary KG, Mandal NN, Mishra S, Mishra R, Sarangi SS, Satapathy AK, et al In-utero sensitization modulates IgG isotype, IFN-γ and IL-10 responses of neonates in bancroftian filariasis. Parasite Immunol. 2014; 36: 485–93. 10.1111/pim.12121 24902619

[14] Mpairwe H, Tweyongyere R, Elliott A. Pregnancy and helminth infections. Parasite Immunol. 2014; 36(8): 328–337. 10.1111/pim.12101 24471654 PMC4260141

[15] Malhotra I, Mungai PL, Wamachi AN, Tisch D, Kioko J, Ouma JH, et al Prenatal T cell immunity to *Wuchereria bancroft*i and its effect on filarial immunity and infection susceptibility during childhood. J Infect Dis. 2006; 193: 1005–1013. 10.1086/500472 16518763

[16] Gillan V, Devaney E. Regulatory T cells modulate Th2 responses induced by *Brugia pahangi* third-stage larvae. Infect Immun 2005; 73: 4034–4042. 10.1128/IAI.73.7.4034-4042.2005 15972491 PMC1168597

[17] Metenou S, Dembele B, Konate S, Dolo H, Coulibaly SY, et al At homeostasis filarial infections have expanded adaptive T regulatory but not classical TH2 cells. J Immunol. 2010; 184: 5375–5382 10.4049/jimmunol.0904067 20357251 PMC3407820

[18] Ateba-Ngoa U, Mombo-Ngoma G, Zettlmeissl E, van der Vlugt LEPM, de Jong S, et al CD4+CD25hiFOXP3+ Cells in Cord Blood of Neonates Born from Filaria Infected Mother Are Negatively Associated with CD4+Tbet+ and CD4+RORct+ T Cells. PLoS ONE. 2014; 9(12): e114630 10.1371/journal.pone.0114630 25531674 PMC4273973

[19] Soboslay PT, Geiger SM, Drabner B, Banla M, Batchassi E et al Prenatal immune priming in *Onchocerciasis-onchocerca volvulus*-specific cellular responsiveness and cytokine production in newborns from infected mothers. Clin Exp Immunol, 1999; 117: 130–137. 10.1046/j.1365-2249.1999.00906.x 10403926 PMC1905471

[20] Christian P, Khatry SK, West KP Jr Antenatal anthelmintic treatment, birth weight, and infant survival in rural Nepal. The Lancet. 2004; 364(9438):981–983. 10.1016/S0140-6736(04)17023-2 15364190

[21] LaBeaud AD, Malhotra I, King MJ, King CL, King CH Do antenatal parasite infections devalue childhood vaccination? PLoS Negl Trop Dis. 2009; 3(5):e442.D, 19478847 10.1371/journal.pntd.0000442 PMC2682196

[22] King CL, Mahanty S, Kumaraswami V, Abrams J S, Regunathan J.Jayaraman K et al Cytokine control of parasite-specific anergy in human lymphatic filariasis: Preferential induction of a regulatory T helper type 2 lymphocyte subset. J Clin Invest 1993; 92:1667–1673. 10.1172/JCI116752 8408619 PMC288325

[23] Mahanty S, Mollis SN, Ravichandran M, Abrams JS, Kumaraswami V, Jayaraman K, et al High levels of spontaneous and parasite antigen-driven interleukin-10 production are associated with antigen-specific hyporesponsiveness in human lymphatic filariasis. J Infect Dis.1996;173: 769–773. 8627051 10.1093/infdis/173.3.769

[24] Achary KG, Mandal NN, Mishra S, Sarangi SS, Kar SK, Satapathy AK, et al Maternal filarial infection: Association of anti-sheath antibody responses with plasma levels of IFN-γ and IL-10. Parasitology. 2013; 140: 598–03. 10.1017/S0031182012002144 23343479

[25] Malhotra I, Ouma JH, Wamachi A, Kioko J, Mangal P, Nizovu M, et al Influence of maternal filariasis on childhood infection and immunity to *Wuchereria bancrofti* in Kenya. Infect Immun. 2003; 71: 5231–37. 10.1128/IAI.71.9.5231-5237.2003 12933869 PMC187356

[26] Malhotra I, Mungai P, Wamachi A, Kioko J, Ouma H, Kazura JW et al Helminth- and Bacillus Calmette-Guérin-induced immunity in children sensitized in utero to Filariasis and Schistosomiasis. J Immunol. 1999; 162: 6843–6848. 10352306

[27] Baumgart M, Tompkins F, Leng J, Hesse M Naturally occurring CD4+ Foxp3+ regulatory T cells are an essential, IL-10-independent part of the immunoregulatory network in *Schistosoma mansoni* egg-induced inflammation. J Immunol. 2006; 176: 5374–5387. 16622005 10.4049/jimmunol.176.9.5374

[28] Hoerauf A, Satoguina J, Saeftel M, Specht S. Immunomodulation by filarial nematodes. Parasite Immunol. 2005; 27: 417–429. 10.1111/j.1365-3024.2005.00792.x 16179035

