# Supplementary material for: Maternal Filarial Infection Influences the Development of Regulatory T Cells in Children from Infancy to Early Childhood
Source: PLoS Negl Trop Dis. 2016 Nov 18;10(11):e0005144. doi: 10.1371/journal.pntd.0005144 (PMC5115651; doi:10.1371/journal.pntd.0005144)
Supplement: S1 Checklist — (DOC) [file pntd.0005144.s001.doc]

STROBE Statement—checklist of items that should be included in reports of observational studies

|  | Item No | Recommendation |
| --- | --- | --- |
| **Title and abstract** | 1 | (*a*) **Maternal filarial infection influences the development of regulatory T cells in children from infancy to early childhood** |
|  |  | (*b*) Children born from filarial infected mothers are comparatively more susceptible to filarial infection than the children born to uninfected mothers. But the mechanism of such increased susceptibility to infection in early childhood is not exactly known. Several studies have shown the association of active filarial infection with T cell hypo-responsiveness which is mediated by regulatory T cells (Tregs). Since the Tregs develop in the thymus from CD4+ CD25^hi^ thymocytes at an early stage of the human fetus, it can be hypothesized that the maternal infection during pregnancy affects the development of Tregs in children at birth as well as early childhood. Hence the present study was designed to test the hypothesis by selecting a cohort of pregnant mothers and children born to them subsequently in a filarial endemic area of Odisha, India . A total number of 49 pregnant mothers and children born to them subsequently have been followed up (mean duration 4.4 years) in an area where the microfilarae ( Mf) rate has come down to <1% after institution of 10 rounds of annual mass drug administration (MDA). Our observations showed that early priming of the fetal immune system by filarial antigens modulate the development of Tregs, which ultimately scale up the production of IL-10 in neonates and creates a milieu for high rate of acquisition infection in children born to infected mothers. To prevent the prenatal immune priming and tolerance supervised therapy can be introduced at the child bearing age of the women, so that they can be free from infection by the time of pregnancy and, thus, decrease the risk of infection during childhood. Implementation of such strategy will help the programme in achieving the target of global elimination of LF by 2020. |
| Introduction | | |
| Background/rationale | 2 | To eliminate LF globally by 2020, WHO has introduced annual mass drug administration (MDA) in different endemic countries since one and half decade. But studies have shown that the infection remains highly prevalent among children below five years of age even after several rounds of MDA . Here question arises what makes these children more susceptible to infection even though infection level has come down below threshold in these endemic areas. It is known that besides host genetics and environmental factors, maternal filarial infection plays some role to increase the susceptibility and outcome of the disease. Since pregnancy and early childhood are critical periods during which the inherited immune system of a child is shaped by the environment, the disease outcome in older age is possibly determined both in in-utero and at birth. But it is not exactly known how in-utero exposures to parasite antigens affect immune responses and ultimately the outcome of disease in early childhood. The mechanism of such effects deserves to be explored since our previous findings suggest supervised therapy before pregnancy can reduce the infection rate among children. Induction of regulatory T cells (Tregs) by pathogen is regarded as one of the mechanism of immune evasion in human. It is known that T cell hyporesponsiveness is associated with the active filarial infection, which is partly mediated by regulatory T cells . The immune suppressive capacities of Tregs are due to production of down regulatory cytokines to inhibit inflammatory responses and facilitate the parasite survival. As the Tregs develop in the thymus at an early stage of the human fetal development from CD4+CD25^hi^ thymocytes, the question arises that whether maternal infection during pregnancy affects the development of Tregs in children during their early life. Here we have made an attempt to find out the answer by evaluating the infection status, level of Tregs and regulatory cytokine IL-10 in a cohort of children born to filarial infected and non infected mothers |
| Objectives | 3 | To investigate the influence of maternal filarial infection on development of T regulatory cell from infancy to early childhood. |
| Methods | | |
| Study design | 4 | This is a cohort study. |
| Setting | 5 | This is a cohort study conducted in District Headquarter Hospital of Khurda, Odisha, India, known to be endemic for filarial (*Wuchereria bancrofti*) infections. Women admitted in O&G Department for delivery from July 2009–July 2011 and are permanent residents of eight adjacent villages have been enrolled for the study conveniently. The district has experienced 10 rounds of MDA since 2004 and reported 0.34% Mf in 2013. Enrolled mothers having healthy full-term children were followed up in a house-to-house visit in the year 2014-15. The mean duration of follow-up was 4.4 years (range, 2-7 years). |
| Participants | 6 | (*a*) The pregnant mothers admitted in the hospital for delivery during 2009-2011 and without any complications, free from other chronic diseases and belongs to this region have been selected for the study. All enrolled mothers have affirmed consumption of anti-filarials distributed during the annual MDA before pregnancy but not during pregnancy since the drugs are not recommended during pregnancy. Enrolled mothers having healthy full-term children were followed up in a house-to-house visit in the year 2014-15. During follow up along with detailed clinical history 1ml of venous blood sample was collected aseptically from each enrolled mothers and her children. On the basis of the availability of the baseline immunological parameters 49 mother-child pairs were identified for follow-up out of 158 mother-newborn pairs enrolled during 2009-2011. |
|  |  | (*b*) Amongst 49 follow up mothers, 28 were CFA positive and 21 were CFA negative at the time of recruitment. The median age of CFA positive mother is 27 years (range, 22-35 yrs) and 25 (range, 21-36 yrs) for CFA negative mothers. All of the study participants were living in rural areas and majority of them (83.3%) were house wives by occupation with primary level of school education (77.5%). |
| Variables | 7 | (i) Mf / CFA status of mother during enrolment and follow up, (ii) ) Mf / CFA status of cord blood and (iii) Mf/CFA status of children born to them during follow-up.(iv) Treg and Il-10 mother during enrolment and follow up, (V) Treg and IL-10 in cord blood, (vi) Treg and IL-10 in children during follow up. |
| Data sources/ measurement | 8* | The data generated by examination of blood collected directly from the participants. Paired cord and maternal blood samples (1ml) were collected at the time of uncomplicated delivery. Venous blood samples were collected from mothers before delivery. Venous umbilical cord blood samples from neonates were collected immediately after birth. The collection of cord blood involved direct aspiration via puncture of the ethanol-sterilized umbilical vein at a site distal to the placenta, to reduce minimum cross-contamination. Maternal and cord blood samples were collected in different sized tubes to avoid the chance of mislabelling. During follow up venous blood samples (1ml) were collected from enrolled mothers and their children along with detailed clinical history. Diagnosis of Mf was done in thick blood smear of peripheral blood collected at night between 20:30 and 22:30 by microscopy and detection of CFA in serum samples using commercial Og4C3 antigen detection assay. Treg expression was measured using flow cytometry and IL-10 was measured using commercially available ELISA kit. |
| Bias | 9 | Only the permanent residents of the study area and admitted in the hospital during delivery has been enrolled in the study and the same participants with subsequently born children were followed up to avoid any sampling bias |
| Study size | 10 | All eligible pregnant women in the study area have been enrolled and those who gave their consent were participated during follow up. |
| Quantitative variables | 11 | Quantitative variables of CFA level , Treg frequency and IL-10 level were analysed using appropriate tests and described in the manuscript. |
| Statistical methods | 12 | (*a)* The statistical analysis was performed using Graph Pad Prism software (version 4). Mann-Whitney test was used to analyze the difference between two groups of unpaired data and Wilcoxon signed rank test for paired data. Fisher's exact test was used to compare the difference of proportions between two groups. Kruskal-Wallis test with the addition of Dunn test was used to analyze the difference between more than two independent groups. The associations between Tregs and IL-10 levels were analyzed using Pearson’s correlation analysis. The level of significance was set at 0.05. |
|  |  | (*b*) Kruskal-Wallis test with the addition of Dunn test was used to analyze the difference between more than two independent groups. |
|  |  | (*c*) No data were missing in the analysis. |
|  |  | (*d*) Twenty one (11.7%) mother out of 179 was excluded because of complication during delivery or infant death or unwillingness. Finally 158 mother-new born pairs were enrolled for the study. During the study period total 109 mother-child pairs have been dropped because they are either non traceable, decline to participate, death of the children, moved out of study area or non availability of immunological parameter. Finally 49 pregnant mothers and their subsequently born children have been followed up during 2014-15 |
|  |  |  |

Continued on next page

| Results | | |
| --- | --- | --- |
| Participants | 13* | (a) A total number of 179 pregnant women admitted to hospital for delivery during July 2009 to July 2011 were evaluated for inclusion in this study. Twenty one (11.7%) of them was excluded and finally 158 mother-new born pairs were enrolled for the study. During follow up total 109 mother-child pairs have been dropped and 49 pregnant mothers and their subsequently born children have been followed up during 2014-15. The mean duration of follow-up was 4.4 years (range, 2-7 years). |
|  |  | (b) At the time of enrolment 21 out of 179 were excluded because of complicated delivery, refused to participate and neonatal / infant death. During follow up total 109 mother-child pairs have been dropped because they are either non traceable, decline to participate, death of the children, moved out of study area or non availability of immunological parameter |
|  |  | (c) A flow diagram of the participants has been provided in the text of the manuscript. |
| Descriptive data | 14* | (a) The study participants were pregnant mothers (n=158) and were living in eight highly filarial endemic rural villages of Odisha (India). At the time of enrolment 11.8% of the mother were microfilariae positive (3–210 per 60μl blood), whereas 44.5% of pregnant mothers were CFA positive (GM: 1925, range: 630–16596). Interestingly, 24.5% of infected mothers have shown transplacental transfer of fiarial antigen to their cord, while none of the cord blood from CFA negative mother was CFA positive. Finally 49 pregnant mothers and their subsequently born children have been followed up during 2014-15. The mean duration of follow-up was 4.4 years (range, 2-7 years). Amongst 49 follow up mothers 28 were CFA positive and 21 were CFA negative at the time of recruitment. All of the study participants were living in rural areas and majority of them (83.3%) were house wives by occupation with primary level of school education (77.5%). Except filarial infection status, no difference was noticed in terms of age in years, multiparity status and educational level among the CFA +ve and CFA-ve mothers during follow-up |
|  |  | (b) No participants with missing data. |
|  |  | (c) *Cohort study*—The mean duration of follow-up was 4.4 years (range, 2-7 years). |
| Outcome data | 15* | *Cohort study*— Out of 158 mother-new born pairs were enrolled for the study only 49 mother –child pair could follow up during this study period. Amongst 49 follow up mothers 28 were CFA positive and 21 were CFA negative at the time of recruitment. Out of 28 children born to the infected mothers, 12 (42.8%) children have acquired filarial infection and become CFA positive. In contrast one of the children (1/21, 4.7%) born to the uninfected mothers has acquired filarial infection and become CFA positive. High level of Treg expression and IL-10 level was marked in children born to infected mother as compared to children born from uninfected mother. A strong positive correlation was observed in children born to infected mother in contrast to children born from uninfected mother. |
|  |  |  |
|  |  |  |
| Main results | 16 | (*a*) A total number of 179 pregnant women admitted to hospital for delivery during July 2009 to July 2011 were evaluated for inclusion in this study. Twenty one (11.7%) of them was excluded and finally 158 mother-new born pairs were enrolled for the study. At the time of enrolment 11.8% of the mother were microfilariae positive (3–210 per 60μl blood), whereas 44.5% of pregnant mothers were CFA positive (GM: 1925, range: 630–16596). Interestingly, 24.5% of infected mothers have shown transplacental transfer of fiarial antigen to their cord, while none of the cord blood from CFA negative mother was CFA positive. During the study period total 109 mother-child pairs have been dropped because they are either non traceable, decline to participate, death of the children, moved out of study area or non availability of immunological parameter. Finally 49 pregnant mothers and their subsequently born children have been followed up during 2014-15. The mean duration of follow-up was 4.4 years (range, 2-7 years).  Amongst 49 follow up mothers 28 were CFA positive and 21 were CFA negative at the time of recruitment. Of the total 28 CFA positive mothers, only 3 were Mf positive at the time of enrollment. All of the study participants were living in rural areas and majority of them (83.3%) were house wives by occupation with primary level of school education (77.5%). Amongst the CFA positive (n=28) follow-up mothers, 18 mothers are still harbouring filarial infection (CFA +ve but Mf –ve) without any clinical symptoms of filariasis, 4 mothers have cleared CFA but have developed acute symptoms of filariasis (episodic attack of fever having lymphangitis of legs/arms) and 6 mothers have cleared CFA without development of any clinical symptoms of filariasis. Whereas none of the CFA negative mothers have acquired filarial infection or developed any clinical sign/symptoms of filariasis.  Out of 28 children born to the infected mothers, 12 (42.8%) children have acquired filarial infection and become CFA positive. In contrast one of the children (1/21, 4.7%) born to the uninfected mothers has acquired filarial infection and become CFA positive. Amongst the infected children 7 children were in the 2 – 4 years of age and 6 children were in 5 – 7 years of age. Out of the 12 CFA positive children 5 were from mothers who continued to be CFA positive where as 7 were from mothers who have cleared CFA. Also none of the children born to either infected or uninfected mother have detectable microfilariae and/or with any clinical signs/symptoms of filariasis.  The expression of Tregs in infected mother–cord pairs was significantly high as compared to mother-cord pairs of uninfected mother. Similarly Tregs cell expression was significantly high in children born to enrolled CFA positive group of mothers in comparison to children born to enrolled CFA negative group of mothers Further we have observed a decreasing trend in the level of Tregs in children born to both infected and uninfected mother as compared to the cord blood. Irrespective of the CFA status of mother at the time of follow-up, Tregs cells were significantly high in mothers who were CFA positive at the time of enrollment compared to enrolled CFA negative mothers. Further, children born to CFA positive mothers had higher levels of Tregs expression than children born to M-Ch- of CFA -ve mother.  At the time of enrollment IL-10 was significantly high in mother as well as cord blood of CFA positive mothers as compared to cord and mother of CFA -ve group. Similarly during follow up significantly high level of IL-10 was observed in CFA +ve mother as well as their children in comparison to CFA –ve mothers and their children. To find out the effect of Tregs cells on IL-10 secretion in infected and uninfected mother as well as their children, a correlation was made during the follow up. No significant correlation was observed between Treg and IL-10 of enrolled CFA+ve and CFA –ve mother during follow up. It is evident that a significant positive correlation exists between IL-10 and Tregs in children of infected mothers. In contrast no correlation was marked between IL-10 and Tregs in children born to CFA negative mothers |
|  |  |  |
|  |  |  |
| Other analyses | 17 | No significant difference in Tregs cell expression was observed among mothers of four different subgroups belonging to CFA positive group. Whereas significantly high Tregs cell expression was observed between these four subgroups of mothers compared to CFA –ve group mothers Children born to four sub-groups of CFA positive mothers showed significant difference among themselves. Further, children born to these four subgroups of mothers had higher levels of Tregs expression than children born to M-Ch- of CFA -ve mother.  However when the comparisons was made between the four subgroups of mothers as well as children belonging to the CFA +ve mothers , no significant difference was observed in IL-10 level among them. But IL-10 level in the subgroup of enrolled CFA positive mothers was significantly high compared to enrolled CFA negative mothers during follow up . More than that IL-10 level was significantly high in children born to all four sub-groups of CFA positive mothers than born to CFA negative mothers |
| Discussion | | |
| Key results | 18 | The current study reveals that maternal *W bancrofti* infection during pregnancy regulates the production of Tregs and IL10 in offspring from infancy to early childhood and children born to infected mothers are at greater risk of acquiring filarial infection than children born to uninfected mothers. The study also emphasizes that in-utero sensitization rather than transplacental transfer of filarial antigen leads to increased susceptibility to filarial infection after birth. |
| Limitations | 19 | The obvious limitation of our study is small sample size corresponding to both children born to infected and uninfected mothers. Albeit by drawing correlation we can interpret that Tregs in offspring from filarial infected mothers influence the IL-10 production as described in adults. When analyzing regulatory T cells, the measurement of transcription factor FoxP3, CD49b and LAG-3 markers for Treg and Tr1 cell population and intracellular FACS antibodies such as IL-10 in IL-10-producing Tregs were not possible due to poor resource which might have been useful to analyze the functional relationships between their number and mechanism of action. |
| Interpretation | 20 | Maternal filarial infection during pregnancy increases the susceptibility of children to infection by immune priming through expression of Tregs as well as regulatory cytokine IL-10. The high incidence of infection among the younger children even after 10 rounds (2014) of MDA in this area is due to high rate of Mf among pregnant women during 2009. While the cause of high Mf rate among the pregnant women might be due to low compliance because of social customs or back to back pregnancy. Hence the present findings relates to a greater impact on mass treatment programs aimed at elimination of transmission of *W bancofti* infection. To prevent the prenatal immune priming and tolerance supervised therapy can be introduced at the child bearing age of the women, so that they can be free from infection by the time of pregnancy and, thus, decrease the risk of infection during childhood. Implementation of such strategy will help the programme in achieving the target of global elimination of LF by 2020. |
| Generalisability | 21 | The results can be generalized beyond the immediate study since many studies carried out in other countries have shown that immunologic memory established by priming of prenatal T cells with antigens that pregnant women encounter through infection persists from gestation to childhood and this might be the cause of high incidence of infection among the younger age children in this cohort as observed by others. Though the focus of immunomodulation during helminth infections has been on IL-10, yet contributions of T regulatory cells appear to be significant because a strong association of Tregs and IL-10 was observed in the present study in children born to filarial infected mother during their early childhood in acquiring infection. |
| Other information | | |
| Funding | 22 | Indian Council Of Medical Research, New Delhi |
